# The Role of Mitochondrial Abnormalities in Diabetic Cardiomyopathy

**DOI:** 10.3390/ijms23147863

**Published:** 2022-07-16

**Authors:** Siarhei A. Dabravolski, Nikolay K. Sadykhov, Andrey G. Kartuesov, Evgeny E. Borisov, Vasily N. Sukhorukov, Alexander N. Orekhov

**Affiliations:** 1 Department of Clinical Diagnostics, Vitebsk State Academy of Veterinary Medicine [UO VGAVM], 7/11 Dovatora Str., 210026 Vitebsk, Belarus; 2 Laboratory of Angiopathology, Institute of General Pathology and Pathophysiology, Russian Academy of Medical Sciences, 125315 Moscow, Russia; drawnman@mail.ru (N.K.S.); andkartuesv@gmail.com (A.G.K.); 3 Petrovsky National Research Centre of Surgery, 2, Abrikosovsky Lane, 119991 Moscow, Russia; borisovevgenij5@gmail.com (E.E.B.); vnsukhorukov@gmail.com (V.N.S.); 4 Institute for Atherosclerosis Research, Osennyaya 4-1-207, 121609 Moscow, Russia; a.h.opexob@gmail.com

**Keywords:** diabetic cardiomyopathy, diabetes mellitus, mitophagy, bioenergetics, Ca^2+^ metabolism

## Abstract

Diabetic cardiomyopathy (DCM) is defined as the presence in diabetic patients of abnormal cardiac structure and performance (such as left ventricular hypertrophy, fibrosis, and arrhythmia) in the absence of other cardiac risk factors (such as hypertension or coronary artery disease). Although the pathogenesis of DCM remains unclear currently, mitochondrial structural and functional dysfunctions are recognised as a central player in the DCM development. In this review, we focus on the role of mitochondrial dynamics, biogenesis and mitophagy, Ca^2+^ metabolism and bioenergetics in the DCM development and progression. Based on the crucial role of mitochondria in DCM, application of mitochondria-targeting therapies could be effective strategies to slow down the progression of the disease.

## 1. Introduction

Cardiomyopathy is a heterogeneous group of diseases of the myocardium associated with diverse conditions. It essential to distinguish primary (e.g., hypertrophic, dilated, restrictive, arrhythmogenic right ventricular, left ventricular non-compaction, ion channelopathies, Takotsubo cardiomyopathy, tachycardia-induced cardiomyopathy, and myocarditis) and secondary (induced by complicating conditions such as hypertension, CAD and valvular heart diseases) cardiomyopathies. However, in many cases, it is not possible to define the clear reason for the development of cardiomyopathy [1]. DCM is usually described by structural and functional abnormalities in patients with DM (diabetes mellitus) without other heart disease risk factors (such as hypertension, coronary artery, and valvular diseases) [2]. Patients with obesity, insulin resistance, and dyslipidaemia are likely to develop a similar metabolism-related cardiomyopathy even without diabetes, which could be referred to as metabolic cardiomyopathy, insulin resistance-induced cardiomyopathy, obesity-related cardiomyopathy, or lipotoxic cardiomyopathy. These patients may not be diagnosed with diabetic cardiomyopathy in the clinical setting because of the absence of diabetes [3]. Number of bio-markers (such as levels of glycated haemoglobin, glucose and low-density lipoprotein, IGFBP7 (Insulin Like Growth Factor Binding Protein 7), TGF-β1 (Transforming growth factor beta 1) and others) could help in DCM diagnosis [4,5]. On the functional level, DCM is associated with diastolic and further systolic dysfunction, cardiac remodelling and myocardial fibrosis, arrhythmia, hypertrophy, and ultimately with clinical heart failure [6].

The development and progression of DCM were associated with inflammation; mitochondrial dysfunction; oxidative stress; impaired cardiac insulin signalling and calcium metabolism; microvascular dysfunction; endoplasmic reticulum stress; endothelial dysfunction; cell death and number of other pathologic conditions [7,8,9,10]. Comparing all DM complications, DM patients have a two-fold greater risk of developing heart failure with DCM accounting for nearly 80% of mortality cases [11].

## 2. Structural and Functional Alterations in DCM

Structural changes in diabetic myocardium are usually assessed by non-invasive reliable and reproducible techniques (such as magnetic resonance imaging and echocardiography), with myocardial fibrosis and hypertrophy as the main hallmarks of DCM [12,13]. While MRI has better sensitivity and capability to provide a more detailed image, it is also more expensive, thus, not so widely used as echocardiography––the main diagnostic tool for the early DCM detection [14,15].

Myocardial fibrosis plays a crucial role in structural reconstruction of the heart in DCM, contributing to the altered LV (left ventricular) function and diastolic dysfunction [16]. Usually, it is described by lower early diastolic filling and increased atrial filling, prolonged isovolumetric relaxation time and supraventricular premature beats, increased LV end-diastolic pressure and diminished LV end-diastolic volume, resulted in the lowered LV ejection fraction and cardiac output [17,18]. On the molecular level, structural and functional changes in the diabetic heart are caused by increased expression of *TGF-β1*, *TSP-1* (thrombospondin-1), MMPs (matrix metalloproteinases) and other related genes, promoting collagen production and increasing content of connective tissue and interrupting normal cardiac function [19,20,21]. Eventually, those changes lead to the loss of cardiomyocytes via the terminal cell death pathway, which could be classified into several forms: autophagy, necrosis, apoptosis, entosis, ferroptosis, oxeiptosis and others [22,23].

Myocardial hypertrophy is the next hallmark of DCM, resulting from the cell-death-mediated loss of cardiomyocytes and subsequent compensative enlargement of the remaining viable cardiomyocytes. On the cellular level, hypertrophy is described with disorganised cardiomyocyte myofiber; on the molecular level, with the over-expression of the key regulatory genes: *β-MHC* (β-myosin heavy chain), *FoxO1* (forkhead box protein O 1), *BNP* (brain natriuretic peptide), *ANP* (atrial natriuretic peptide), adiponectin signalling pathway, and others [24,25]. Despite extensive and meticulous research, the exact molecular mechanism of cardiac hypertrophy pathogenesis is not fully understood.

In the further steps of the DCM development, myocardial fibrosis and hypertrophy promote progression of cardiomyocyte stiffness and subsequent diastolic dysfunction. Mechanically, such an effect could be explained by impaired insulin signalling, which affects GLUT4 (glucose transporter type 4) and Ca^2+^ pumps, and resulted in reduced glucose intake and increased intracellular level of Ca^2+^ in cardiomyocytes [26]. Ca^2+^ overload triggers mitochondrial permeability transition pore opening and leads to cardiomyocytes death, while the restoration of Ca^2+^ levels with MCU (mitochondrial calcium uniporter) expression reduces the infarcted area in diabetic heart and increases glucose utilization (which manifested by improved mitochondrial respiratory function and ATP production) [27]. Simultaneously, disturbed insulin signalling leads to reduced activity of eNOS (endothelial nitric oxide synthase), which also affects Ca^2+^ metabolism and reduces Ca^2+^ intake by sarcoplasm [28,29]. Altogether, such pathophysiological abnormalities lead to cardiac stiffness, impaired cardiac relaxation and diastolic dysfunction as the early DCM manifestation.

## 3. Mitochondrial Dynamics in DCM

Mitochondria capacity and functions are regulated via their biogenesis, dynamics and recycling. PGC1α (peroxisome proliferator-activated receptor gamma coactivator 1-alpha) is the major regulator of mitochondrial biogenesis, which could be triggered by development signals, internal and environmental stimuli [30]. Damaged and dysfunctional parts of mitochondria are separated from healthy parts during mitochondrial turnover—cycles of fission (split) and fusion (merge). Mitochondrial fission and fusion are regulated by *DNM1L* (dynamin 1 like)/*FIS1* (fission, mitochondrial 1) and *MFN1*, *MFN2* (mitofusin 1 and 2)/*OPA1* (optic atrophy protein 1) genes, respectively [31]. Furthermore, damaged parts of mitochondria are fused with lysosome and digested during the process of a specialised form of mitochondrial autophagy––mitophagy. In general, mitophagy regulation could be classified as conventional PINK1/Parkin (PTEN Induced Kinase 1/Parkin RBR E3 Ubiquitin Protein Ligase)-mediated and alternative Parkin-independent mitophagy [32]. Healthy parts of mitochondria are returned to the mitochondrial network to continue normal functioning [33,34]. Mitophagy is crucial for many cellular processes, normal organism functioning, and development; its dysregulation is associated with neurodegenerative diseases, cancer, inflammation and other pathological conditions [35,36]. The heart relies on the ATP generated by mitochondria via aerobic metabolism (from the oxidation of glucose and FAs (fatty acids)). Therefore, any dysregulation of mitochondrial cellular functions eventually leads to a decrease in ATP and increase of ROS (reactive oxygen species) production, which contributes to the development of different cardiovascular diseases [31,37]. In normal physiological conditions, the balance between mitochondrial fusion and fission keeps mitochondrial functions at the level required by the current cellular needs. However, fission/fusion imbalance leads to a significant rise in ROS release and reduction of ATP production, stimulating cell death and further disease progression [38,39].

**Figure 1:**
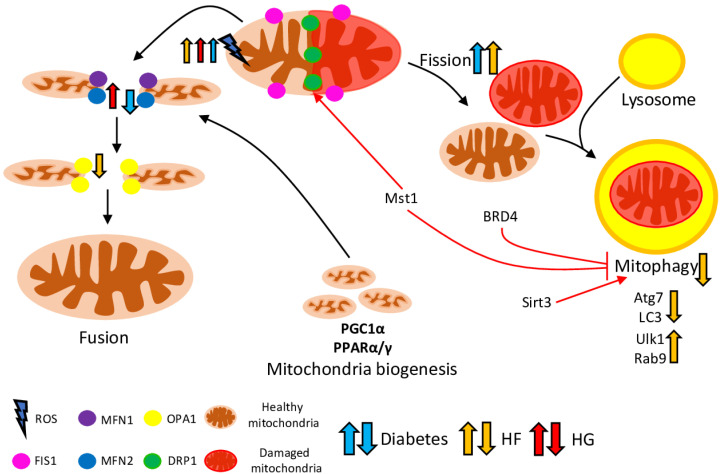
Interconnections between mitophagy, mitochondrial dynamics, and mitochondrial biogenesis in DCM. Damaged and malfunctional mitochondria are isolated via mitochondrial fission, merged with lysosome, and digested through mitophagy. Healthy parts of mitochondria are returned to the mitochondrial network through mitochondrial fusion. Mitochondrial fission is mediated by Drp1 and Fis1, fusion––Mfn1 and 2, OPA1, while conventional mitophagy is guided by Atg7 (Autophagy-Related Protein 7) and LC3 (Autophagy-Related Ubiquitin-Like Modifier LC3 A), and alternative––by Ulk1 (unc51 like kinase 1)- and Rab9 (Ras-related protein Rab-9)-dependent mechanism. The adequate mitochondrial pool is supported via generation of new mitochondria, which is regulated by transcription factors (such as PPARα/γ) and cofactors (such as PGC1α), and sustained the supply of energy to the myocardium. The imbalance between mitochondrial dynamics, mitophagy, and mitochondrial biogenesis leads to metabolic dysregulation and cardiomyocytes’ damage. The effects of the diabetes, high fat (HF), and high glucose (HG) conditions on the key players are depicted with blue, yellow and red arrows, respectively.

Recently, it was shown that hearts from diabetic mice have a surplus rate of mitochondrial fission, resulting in a decrease of mean mitochondrial size and number of mitochondria per μm^2^. In addition, the expression level of *MFN2* was significantly decreased, while levels of other dynamics-related proteins (Opa1, Mfn1, Fis1 and Drp1) were not changed (Figure 1). Furthermore, over-expression of Mfn2 in diabetic hearts reduces ROS production, restores mitochondrial membrane potential, and normalises fission. Mfn2 expression could be regulated via its positive regulator PPARα, which directly binds to an Mfn2 promoter [40]. Similarly to Mfn2 over-expression, treatment of HFD-induced prediabetic mice with necrostatin-1, an inhibitor of cell death and inflammation receptor RIPK1, with known cardioprotective properties [41], effectively enhanced Mfn2 protein levels, with no alterations in Mfn1 and Opa1 protein levels, thus increasing mitochondrial fusion and alleviating mitochondrial dysfunction. In addition, necrostatin-1 effectively reduced cardiac dysfunction, cardiac autonomic imbalance, and blood pressure in prediabetic rats [42].

High glucose levels could induce mitochondrial dysfunction and cell death in rats’ atrial cardiomyocytes. In in vitro conditions, hyperglycaemia increases ER (endoplasmic reticulum) stress, mitochondrial ROS levels, and Mfn2 levels. On the contrary, for the results from the hearts of diabetic mice, Mfn2 silencing decreases Ca^2+^ transfer from ER to mitochondria under ER stress conditions, ameliorating mitochondrial ROS production and oxygen consumption, thus preventing cells from mitochondrial dysfunction and protecting from ER-mediated cell death [43,44]. Similarly, hyperglycaemia increases mitochondria–ER contact and Ca^2+^ levels in the ER and mitochondria of cardiomyocytes. However, Mfn2 silencing reduces mitochondria-ER cooperation, Ca^2+^ concentrations in both ER and mitochondria, mitochondrial ROS production, ER stress and mitochondria-mediated apoptosis, preserving mitochondrial function and cardiomyocyte viability under hyperglycaemic conditions [45].

Drp1 (Dynamic relative protein 1) regulates mitochondrial fission by changing its level of phosphorylation. As such, mitochondrial fission is driven via S616 phosphorylation by ERK (extracellular signal-regulated kinase), while calcineurin induces S637 dephosphorylation and mediates Ca^2+^-induced mitochondrial fission [46]. Recent research suggests that, besides Drp1, the Orai1 (Ca^2+^ release-activated calcium channel protein 1) calcium channel plays an important role in cardiomyocytes Ca^2+^ metabolism and DCM development. Cardiac hypertrophy and impaired cardiac function, accompanied by increased mitochondrial fission and intracellular Ca^2+^ overload, were described in diabetic rats on HGD (high glucose diet). However, application of Drp1 inhibitor prevents HGD-induced cardiomyocyte hypertrophy by reducing Drp1 S616 and increasing S637 phosphorylation. Likewise, Drp1 activity could be suppressed with Orai1 inhibition, which also alleviates HGD-induced mitochondrial dysfunction and cardiomyocyte hypertrophy, suggesting Drp1 as a downstream target of Orai1-mediated Ca^2+^ influx [47].

Recently, the role of Mst1 (Mammalian sterile 20-like kinase 1), one of the crucial components of the Hippo signalling pathway involved in regulating cell apoptosis and cell proliferation, in the development of cardiovascular disorders was shown [48]. Mst1 regulates mitochondrial fission via upregulating Drp1 expression and its recruitment to the mitochondria, stimulating S616 phosphorylation and D637 dephosphorylation. Furthermore, Mst1 knockdown in diabetic mice inhibits mitochondrial fission and mitigates LV remodelling and cardiac dysfunction, thus preventing the DCM development. In addition, Drp1 knockdown prevents the effects of Mst1 on mitochondrial fission and DCM development, thus supporting the proposed Drp1-mediated mechanism [49].

Cardiomyocyte-specific over-expression of *LACS1* (long chain acyl-CoA synthetase 1), used as a mice model system with increased cardiac FA uptake, resulted in dramatically remodelled mitochondrial network and abnormal mitochondrial dynamics. Long-term exposure of transgenic mice to FA leads to increased mitochondrial ROS production, fission rate, and loss of the mitochondrial reticulum. On the molecular level, high FA induces surplus ubiquitination of AKAP121 (A-kinase anchor protein), a crucial regulator of mitochondria physiology [50], which further reduces S637 phosphorylation of Drp1 and enhances Opa1 proteolysis. Therefore, cardiac lipid overload induces increased mitochondrial ROS generation and causes alteration in post-translational modifications of Drp1 and Opa1, which leads to dysregulation of mitochondria fusion and fission, thus contributing to cardiac dysfunction in diabetes and obesity [51].

In total, DM-associated pathological conditions (such as hyperglycaemia and lipid overload) cause increased mitochondrial ROS generation and post-translational modifications of mitochondrial proteins that regulate mitochondrial dynamics, which further lead to imbalanced mitochondrial dynamics and contribute to DCM development (Figure 1). Therefore, further research is required to investigate the role of mitochondrial dynamics in DCM development and to comprehend its potential for DCM treatment.

## 4. Mitophagy

Mitophagy is a specialised form of autophagy, crucial for normal organism functioning and development. Normally, nutritional deficiency and cell senescence stimulate mitophagy, targeting damaged and malfunction mitochondria to fuse with lysosomes to degrade. Mice in prediabetic conditions were characterised with a mild diastolic dysfunction, increased SR mitoROS production, increased number of myocardial lipid droplets, elevated LV mass and wall thickness, increased expression of the *MFN2* and decreased expression of a mitophagy marker *BNIP3* (Bcl-2/adenovirus E1B 19-kDa protein-interacting protein 3). On the contrary, no sign of cardiac hypertrophy or fibrosis was observed. Those results suggest that abnormal mitochondrial dynamics and mitophagy are the primary metabolic derangements responsible for initial cardiac pathological changes [52]. Additionally, the crucial role of mitophagy was confirmed in experiments on mice with HFD-induced diabetes, where the mitophagy rate increases after three weeks of HFD feeding. Deletion of atg7 or Parkin resulted in impaired mitophagy, increased lipid accumulation, exacerbated diastolic dysfunction and induced systolic dysfunction. On the contrary, mitophagy activation with injection of Tat-Beclin1 attenuated mitochondrial dysfunction, decreased lipid accumulation, and protected against cardiac diastolic dysfunction during HFD feeding, suggesting its protective role against HFD-induced diabetic cardiomyopathy [53].

Besides the role in regulating mitochondrial dynamics, Mst1 was shown to inhibit mitophagy in a SIRT3-Parkin-dependent way. Sirt3 (NAD-Dependent Deacetylase Sirtuin-3, Mitochondrial) is a vital member of the class III histone deacetylases, known to eliminate mitoROS, inhibit apoptosis, and prevents the formation of cancer cells. Mst1 deficiency stimulated mitophagy in DCM and alleviated DCM-associated phenotype in an HGD-induced diabetic mice model. However, positive effects of Mst1 knockdown were abolished in Parkin^−/−^ and Sirt3^−/−^ mice [54]. Another research supports the role of Sirt3 in DCM via the Foxo3A-Parkin signalling pathway. Sirt3 knockout aggravated effects of streptozotocin-induced diabetes in mice: suppressed autophagy and mitophagy, cardiomyocyte apoptosis and mitochondrial injury, cardiac dysfunction, and interstitial fibrosis. At the same time, the deacetylation of Foxo3A and expression of Parkin were decreased. In vitro Sirt3 overexpression activated autophagy and mitophagy, inhibited mitochondrial injury and cardiomyocyte apoptosis, normalised Foxo3A deacetylation and Parkin expression [55]. Because of the crucial role of Sirt3 and Mst1 regulation of mitochondrial dynamics and mitophagy, further studies required to better understand their involvement in the development of diabetic cardiomyopathy and potential use as therapeutic targets for DCM treatment [56].

Recent research proved that HFD itself suppressed mitophagy and the accumulation of damaged mitochondria in the heart of obese/diabetic mice. Experiments with cardiac-specific deletion of ACC2 (acetyl-CoA carboxylase 2), a vital enzyme, catalysing the carboxylation of acetyl-CoA to malonyl-CoA, which could inhibit carnitine-palmitoyl-CoA transferase I, the rate-limiting step in FA uptake and oxidation by mitochondria, suggested that enhanced cardiac FAO does not cause cardiomyopathy in non-obese mice. On the contrary, ACC2 knockout in mice on HFD increased cardiac FAO, preventing HFD-induced downregulation of Parkin and subsequent mitophagy suppression, mitochondrial dysfunction and pathological heart remodelling, thus protecting from DCM [57].

The epigenetic mechanism is also involved in regulation of mitochondrial energy production and mitophagy, and could be used for DCM treatment. *BRD4* (Bromodomain Containing 4) is an ubiquitously expressed chromatin regulator protein, which plays a pivotal role in cardiomyocyte specific mitochondrial energy production and homeostasis [58]. Application of selective BRD4 inhibitor JQ1 mitigates DCM, heart failure, and cardiac hypertrophy [59,60]. Interestingly, diabetic mice hearts have increased the level of BRD4, which inhibits PINK1/Parkin-mediated mitophagy and causes further accumulation of damaged mitochondria, impairment of cardiac function and heart remodelling. Treatment with a JQ1 inhibitor improves mitochondrial and heart function in a Pink1-dependent way and prevents HFD-induced DCM, supporting BRD4 inhibition as a valuable strategy for DCM treatment [61].

Interestingly, chronic HFD consumption inactivates conventional mitophagy (mediated by the Atg7 and LC3), whereas stimulates alternative (Ulk1 (unc51 like kinase 1)- and Rab9 (Ras-related protein Rab-9)-dependent mechanism of mitophagy. Thus, 24 weeks of HFD consumption upregulated both Ulk1 and Rab9 in the mitochondrial fraction, while cardiac-specific ulk1 knockout and Rab9 knock-in mice showed impaired mitophagy and both diastolic and systolic dysfunction (Figure 1). On the contrary, heart-specific Rab9 over-expression increased mitophagy and protected against cardiac dysfunction during HFD consumption. Apparently, alternative mitophagy mechanisms do not sustain mitochondrial quality under prolonged HFD burden; however, they could be therapeutically enhanced to obstruct DCM progression in patients with obesity and diabetes [62].

In summary, mitophagy plays an important role in the prevention of cardiac mitochondria dysfunction, cardiac hypertrophy, diastolic dysfunction, and lipid accumulation in the hearts of diabetic mice (Figure 1). Although the exact molecular mechanism of the cardiac mitophagy regulation is still unknown, stimulation of conventional and alternative mitophagy effectively protects the heart against DCM and provides a rationale to develop new mitophagy-targeted therapeutic approaches.

## 5. Mitochondrial Unbalanced Calcium Homeostasis

**Figure 2:**
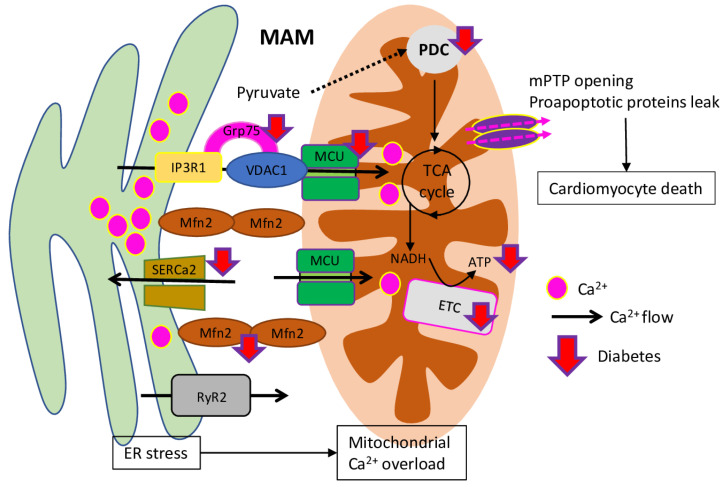
Influence of diabetes on mitochondrial Ca^2+^ homeostasis. Diabetes inhibits key players, indicated by red arrows. Mitochondria and ER/SR are linked by several proteins resulting in optimised Ca^2+^ flows between these two organelles. GRP75 and Mfn2 serve as anchoring proteins, narrowing the MAMs (mitochondrial associated membranes) space between IP3R2/VDAC and RyR2. The mPTP opening can be induced by increased ROS, Ca^2+^ or decreased ΔΨm, which is resulting in the release of the mitochondrial content including pro-apoptotic proteins. Abnormal expression of proteins of the MCU results in diminished Ca^2+^ uptake with reduced [Ca^2+^]_m_. This [Ca^2+^]_m_ reduction contributes to the occurrence of mitochondrial dysfunctions, in particular, a decrease in the PDC activity, followed by decreased mitochondrial bioenergetics. Consequently, the insufficient ATP content cannot match the energy demand for the cardiomyocyte contraction required for the normal functioning of the heart.

The mitochondrial calcium concentrations play a critical role in cardiac excitation-contraction coupling. In particular, buffering capacity is facilitating signalling crosstalk between mitochondria and ER/SR (sarcoplasmic reticulum), mitochondria, and plasma membrane. Mitochondrial Ca^2+^ regulates glucose oxidation (via activation of PDC (pyruvate dehydrogenase complex)) and enhances ATP formation (via activation of mitochondrial complexes I, III, IV, and V) (Figure 2). It is known that free mitochondrial Ca^2+^ ([Ca^2+^]_m_) concentration, as a key signalling molecule for mitochondrial energetic metabolism, is decreased in cardiomyocytes from diabetic hearts [63]. During myocardium excitation contraction coupling, Ca^2+^ gets into the cytoplasm via voltage sensitive LTCCs (L-type calcium channels) after sarcolemma depolarisation, triggering Ca^2+^ release from the SR. During the diastolic process, Ca^2+^ is pumped back to SR, accompanied by the excess Ca^2+^ pumped out through Ca^2+^ pump on the plasma membrane and sarcolemma Na^+^/Ca^2+^ exchanger [64,65]. However, in DCM, the Ca^2+^ transporters and Ca^2+^ transfer between mitochondria, ER and SR calcium homeostasis is disrupted, which resulted in increased duration of action potential and prolonged diastolic relaxation time [10].

As it was shown, mouse cardiac myocytes under hyperglycaemic conditions have reduced [Ca^2+^]_m_ and protein level of MCU (mitochondrial Ca^2+^ uniporter)––the channel responsible for mitochondrial Ca^2+^ uptake in mitochondria and regulation of Ca^2+^-dependent mitochondrial metabolism. However, MCU over-expression normalises the [Ca^2+^]_m_ level, which is responsible for subsequent reduction of oxidative stress, apoptosis and FAO (fatty acid β-oxidation), increase of glucose oxidation, PDH (pyruvate dehydrogenase) activity, and mitochondrial membrane potential, thus reversing hyperglycaemia-induced metabolic alterations [66]. In addition, those results were confirmed in vivo in streptozotocin (STZ)-induced diabetic mice, where MCU normalisation besides improved mitochondrial function and Ca^2+^ handling, cardiac energetic metabolism and both cardiac myocyte and heart function [26]. Similar results were obtained on db/db diabetic mice, where over-expression of *MICU1* (mitochondrial calcium uptake 1), the regulatory subunit of MCU, inhibits the development of DCM (through the enhanced cardiac function, reduced myocardial fibrosis and cardiac hypertrophy). In vitro data suggest that hyperglycaemia and hyperlipidaemia inhibited the expression of *MICU1*-regulating transcription factor Sp1, while *MICU1* over-expression increases mitochondrial Ca^2+^ uptake and inhibits ROS production through enhancing the NADPH-dependent antioxidant system [67].

Recent research has showed that, besides mitochondrial Ca^2+^ entry through the MCU uniporter, the dysregulation of reticulum–mitochondrial Ca^2+^ coupling is also involved in the DCM development. Such hotspot for Ca^2+^ fluxes from ER to mitochondria are called MAM (mitochondria-associated membranes) and formed primarily by IP3R/Grp75/VDAC (Inositol 1,4,5-Trisphosphate Receptor Type 3/Stress-70 Protein, Mitochondrial/Voltage-dependent anion-selective channel protein 1) Ca^2+^ channelling and tethering complex [68]. Diabetic mice on a high-fat high-sucrose diet with cardiac insulin resistance, hypertrophy, diastolic dysfunction, fibrosis and lipid accumulation, on the molecular level, were represented with a decreased IP3R–VDAC interaction and a reduced IP3-stimulated Ca^2+^ transfer to mitochondria, while the MCU function, cytosolic Ca^2+^ transients and reticular Ca^2+^ level were not changed. In total, described alterations lead to a decreased mitochondrial bioenergetics and reduced cell contraction. However, switching back to a standard diet could reverse such Ca^2+^ miscoupling, restore normal cardiac and mitochondrial functions, and prevent DCM development [69].

Additionally, CaMKII (Ca^2+^/calmodulin kinase II), the key mediator of many of the second messenger effects of Ca^2+^, and RyR2 (ryanodine receptors), which mediates the release of Ca^2+^ from the cardiac muscle SR into the cytoplasm and, therefore, plays a crucial role in triggering cardiac muscle contraction, are involved in diabetic impaired Ca^2+^ handling, cardiac arrhythmias and apoptosis [70]. Mice with fructose-rich diet-induced prediabetic conditions have increased CaMKII activity, which resulted in SR Ca^2+^ leak by RyR2 activation, enhanced SR-mitochondria tethering, and increased mitochondrial fission and H_2_O_2_ production, arrhythmia. However, such changes were prevented in AC3-I mice with myocardial-targeted CaMKII inhibition [71]. Recent research has established the connection between diabetes, inflammation, and arrhythmias. As it was shown in the HFD-induced DM mice model, increased mitochondrial ROS is accompanied with elevated levels of cardiac IL-1β (interleukin), increased oxidation of SR Ca^2+^ channel RyR2, and subsequent Ca^2+^ leakage through it and risk of arrhythmia. However, application of IL-1β and mitoROS inhibitors stabilises RyR2 oxidation, reducing Ca^2+^ leak and diabetic arrhythmia risk, thus suggesting involvement of the innate immune mechanisms in the DCM development [72]. Similar results were obtained on the PPARγ mice with cardiac lipid overload, where the level of mitochondrial ROS was connected with increased RyR2 oxidation. Furthermore, surplus RyR2 oxidation resulted in increased calcium transient amplitude and SR calcium, and, subsequently, in increased spontaneous contractions and spark frequency. However, application of mitochondrial-targeted anti-oxidants normalises Ca^2+^ handling and heart rhythm, thus suggesting their high potential as a therapy to prevent arrhythmia and sudden cardiac death in obese and diabetic patients [73].

Interestingly, trace elements, such as Zn and Cu, also involved in cell signalling, glucose homeostasis, mitochondrial function, and other processes, related to DM progression and DCM. For example, application of zinc sulphate effectively ameliorates cardiac hypertrophy, inflammation, oxidative damage and fibrosis of transgenic OVE mice T1DM model [74]. Treatment of T2DM model ZDF (Zucker diabetic fatty) rats with zinc and acetylsalicylic acid (as bis(aspirinato)zinc(II)-complex Zn(ASA)2) resulted in normalisation of left-ventricular diastolic stiffness, collagen content, reduction of cardiomyocyte DNA-fragmentation, and nitro-oxidative stress, presumably, acting via upregulation of AKT (Protein Kinase B), a crucial regulator of many cellular functions (such as cell proliferation, metabolism, survival, angiogenesis, and others) [75,76]. Furthermore, gestational diabetes-associated foetal myocardial damage could be effectively restored with Zn sulfate, which normalises myofibrils architecture, decreases apoptosis and the levels of ROS, replenishing the antioxidant pool [77]. Similarly, Cu(II)-selective chelator, triethylenetetramine, improves cardiac pump function, levels of the copper chaperones (Cox17 and Cox11), enzymatic activity of mitochondria-resident copper-enzymes (cytochrome c oxidase and superoxide dismutase 1), and normalises *PGC1α* expression in LV tissues of STZ-induced diabetic rats [78]. However, while many studies have proved an important role of several trace elements in the DM and DCM pathogenesis, those elements are toxic in excessive amounts [79].

In total, impaired mitochondrial Ca^2+^ handling plays the key role in DCM. However, many other factors and trace elements contribute to the development of diabetes-associated pathological conductions. Impaired mitochondrial Ca^2+^ metabolism affects other signalling pathways and interactions with other organelles. Therefore, more profound investigation is required to define the exact molecular mechanisms and signalling pathways involved in Ca^2+^ and trace elements regulation and DCM development.

## 6. Mitochondrial Energy Metabolism in DCM

**Figure 3:**
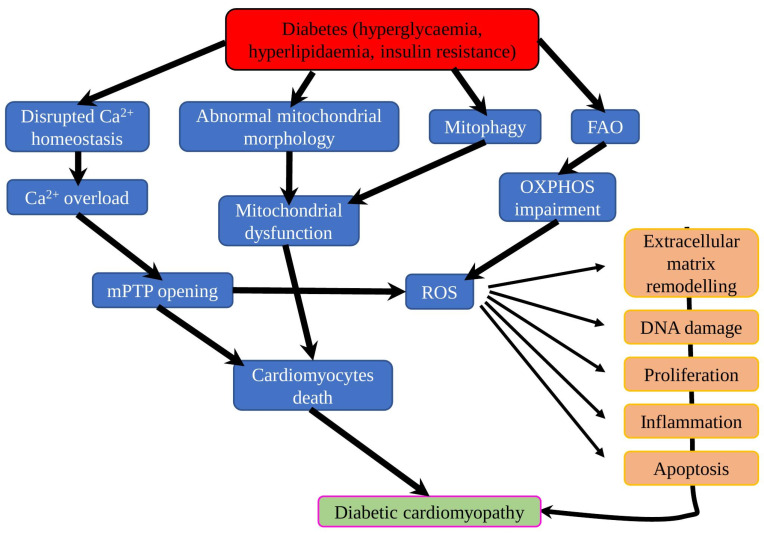
Involvement of mitochondrial dysfunction in diabetic cardiomyopathy. In the diabetic condition, ATP is mostly generated from FAO instead of glucose, which leads to OXPHOS impairment and generates more ROS. Disrupted Ca^2+^ handling leads to mitochondrial Ca^2+^ overload and mPTP opening, which causes mitochondrial respiratory dysfunction, increased ROS production and a number of consequential effects (such as increased DNA damage, apoptosis rate, inflammation and others). Mitophagy plays a protective role, removing damaged mitochondria and attenuating mitochondrial dysfunction.

Under physiological conditions, cardiomyocytes require a high amount of energy, which is mostly produced by mitochondria via the FAO (constitutes about 70%) and the remaining part originating from other substrates (ketone bodies, glucose, amino acid and lactate). However, compared to glucose, energy production from FAs requires 12% more oxygen to earn the same amount of ATP [80]. The diabetic heart is characterised by decreased glucose oxidation and increased FAO, which resulted in increased oxygen consumption, respiratory dysfunction in mitochondria and hypoxia aggravation in myocardium with microangiopathy (Figure 3) [81]. As it was shown in the mice fructose-induced T2DM model, early diabetic hearts have evidence of oxidative stress, accompanied with increased cellular and mitochondrial FA uptake, and increased FAO, but also reduced LCAD (Acyl-CoA Dehydrogenase Long Chain) activity and mitochondrial mass. In addition, the activity of SIRT1, a crucial regulator of FA metabolism, was decreased, confirming its important role in cardiac metabolic adaptations via FAO modulation [82]. In the later stages, more severe effects of DMC appeared, for instance, STZ-induced DM mice on HFD for 26 weeks had increased myocardial fibrosis and LV diastolic dysfunction, elevated LV superoxide levels, decreased mitochondria area, increased levels of mitochondrial complex III, and complex V protein abundance, while reducing complex II oxygen consumption [83].

Recent research revealed that reduced cardiac glucose uptake and oxidation is a protective metabolic adaptation, protecting mitochondria from glucotoxicity. Transgenic non-diabetic mice with inducible cardiomyocyte-specific expression of the *GLUT4* had decreased mitochondrial ATP generation and evidence of diastolic dysfunction. On the contrary, diabetic mice had increased O-GlcNAcylation of the transcription factor Sp1, many electron transport chain subunits and other mitochondrial proteins, which resulted in aggravated mitochondrial oxidative dysfunction, suggesting mitochondria as a main target of glucotoxicity [84].

Results from the T1DM-induced rat model suggested increased heart mitochondrial mass, volume and number per unit area, whereas the mean area of each mitochondrion was decreased compared to control rats. In addition, diabetic rats’ heart mitochondria had higher production of H_2_O_2_ and NO while a lower ATP production rate. Apparently, increased H_2_O_2_ and decreased energy production triggered a compensatory mechanism to stimulate the de novo mitochondria biogenesis via upregulation of PGC1α, the major mitochondria biogenesis regulator, which aimed to restore normal energy supply in diabetic pathological conditions [85]. Further research shows that complex I dysfunction reduces ATP production, and the increase in H_2_O_2_ and NO production could be considered as early subcellular signals of cardiac mitochondrial dysfunction, while PGC1α-mediated de novo synthesis of mitochondria is activated on the later stage of DCM [86,87]. Interestingly, other researchers have proved that *PGC1α* expression is reduced because of endogenous accumulation of nitric oxide synthase inhibitor ADMA (asymmetric dimethylarginine) in both T1DM and T2DM rat models, thus suggesting it as a novel therapeutic target to prevent or treat DCM [88].

Interestingly, the presence of DM-related diseases (such as CVDs and obesity) could also affect cardiac mitochondrial energetics and ETC (electron transport chain) efficacy. As it was recently shown, atrial appendages from patients with diabetes and AF (atrial fibrillation) had impaired function of complex I and II, aggravated ETC supercomplex assembly and increased oxidative damage. On the contrary, such abnormalities were not observed in patients with diabetes but without AF [89]. Similarly, mitochondria isolated from right atrial appendage tissues patients with coronary heart disease with/without diabetes had depressed complex respiration CHD patients regardless of the presence of diabetes, while complex II was repressed only in CHD patients with diabetes. Those results are suggesting a more significant mitochondrial dysfunction in the presence of diabetes [90]. In another study with similar set-up, mitochondria isolated from right atrial appendage tissues of DM patients with CHD had decreased oxygen consumption rate for fatty acid substrate and impaired complex I activity. Moreover, mtDNA copy number, levels of ETC complex components, FA metabolism and regulation proteins were unchanged between diabetic and non-diabetic patients [91].

Surprisingly, maternal HFD and/or diabetes could cause cardiac dysfunction in offspring through metabolic stress and impairment of mitochondrial function. Glycolytic and respiratory capacity was impaired in DM-exposed offspring, while HFD-exposed offspring had evidence of mitochondrial dysfunction, increased mitochondrial copy number and rate of lipid peroxidation. Offspring exposed to both DM and HFD had severe symptoms similar to adult DCM, such as accumulation of cardiac lipid droplet and diastolic/systolic cardiac dysfunction [92]. Interestingly, further research suggested different effect of maternal diabetes on male and female offspring of weaning and adult age. As such, only adult male offspring had an elevated level of autophagy and decreased respiration of mitochondrial complex I and II. However, all groups (age and gender) had normal expression levels of antioxidative enzymes (glutathione peroxidise and mitochondrial superoxide dismutase), suggesting absence of oxidative stress [93].

In total, the presence of DM in combination with obesity, CVD and other co-morbidities gradually decreasing efficacy of mitochondria energy production, affecting mostly complexes I and II, finally resulted in mitochondrial dysfunction and DCM. However, mitochondria have several compensatory mechanisms, aiming to restore mitochondrial functions and normal energy supply, suggesting that those mechanisms could be targeted to improve mitochondrial function in DCM treatment or prevention.

## 7. Mitochondria-Targeting Approach in DCM Treatment

The primary role of mitochondria in DCM pathogenesis was recently established in experiments on PGDM (pre-gestational diabetes mellitus)-exposed rats. PGDM-exposed offspring had cardiac dysfunction at birth, accompanied by low ATP generation, high lipid peroxidation, and high apoptosis rate under metabolic stress. However, transfer of mitochondria isolated from normal rat myocardium to PGDM-exposed rat cardiomyocytes resulted in a sex-specific stimulation of oxygen consumption, ATP production, mitophagy stimulation and reduction of stress-induced apoptosis [94].

Because of the crucial role of mitochondria in DCM pathogenesis and development, several specific mitochondria-targeting therapeutics for DCM treatment and prevention have been invented and successfully tested. For example, mitochondrial ROS inhibitor mito-TEMPO reduced adverse cardiac changes and mitigated myocardial dysfunction in both T1DM and T2DM mice models [95]. Similarly, MitoGamide, the scavenger of methylglyoxal, a mitochondria-damaging by-product of glycolysis and a reactive carbonyl species, showed cardioprotective properties in tests on diabetic Akita mice [96], where it significantly improved the E/A ratio—the key indicator of diastolic dysfunction in the development of diabetes-induced HFpEF (heart failure with preserved ejection fraction) [97]. Application of alisporivir, a mitochondria-targeted non-immunosuppressive analogue of cyclosporin A, which acts as a selective inhibitor of the MPT pore opening, was investigated in the heart of DM-induced mice. Alisporivir effectively restored the blood glucose level and the indicator of heart rate, preventing mitochondrial swelling and ultrastructural alterations in DM mice cardiomyocytes. Simultaneously, alisporivir stimulated the mitophagy rate in the heart tissue, thus activating removal of damaged mitochondria [98]. Similarly, hydrogen sulfide (H_2_S) was shown to provide mitochondria-mediated cardioprotective effects in STZ-induced T1DM rats. Particularly, exogenous H_2_S treatment leads to decreased expression of mitochondrial apoptotic proteins, cyt C, mPTP opening, normalised MFN2 expression and cardiac function [99]. FGFs (fibroblast growth factors) are involved in the regulation of metabolism on many levels. Primarily, FGF21 was used to correct metabolic dysfunction associated with diabetes and obesity, while cardioprotective properties were discovered and explored later (reviewed in [100,101]). FGF21 long-term supplementation improved heart rate variability and left ventricular function, attenuated insulin resistance and activated anti-apoptotic and cardiac mitochondrial FAO signalling pathways in obese, insulin-resistant rats [102]. Mechanically, FGF21 prevented T2DM lipotoxicity-induced cardiomyopathy through antioxidative (AMPK–AKT2–NRF2-mediated pathway) and lipid-lowering (AMPK–ACC–CPT-1-mediated pathway) effects in the diabetic heart [103]. Similarly, FGF1 treatment reduced mitochondrial fragmentation and ROS generation, cytochrome c leakage and enhanced mitochondrial respiration rate and β-oxidation, which resulted in restored cardiac function [104].

To sum up, application of mitochondria-targeted treatments provides wide physiological effects and stimulates favourable metabolic processes, confirming its high potential in the treatment of DCM and other metabolic disorders [105].

## 8. Zinc Supplementation in DM Treatment and Cardioprotection

It is known that Zn^2+^-associated signalling pathways are involved in the development of diabetic cardiomyopathy and other heart diseases. Mitochondria coordinate [Ca^2+^]_i_ and [Zn^2+^]_i_ homeostasis, which affect cellular ROS production, mitochondria ultrastructure and efficiency [106,107]. The role of Zn^2+^ homeostasis in DM and obesity-induced cardiac remodelling, inflammation, dysfunction and other cardiovascular diseases and complications was covered in several recent reviews [108,109,110]. Here, we wish to describe several recent randomised trials designed to investigate the effect of zinc supplementation on DM progression.

Zn supplementation (20 mg daily) of subjects with pre-diabetes in a randomized, double-blind, placebo-controlled Phase II clinical trial in Sri Lanka for 12 months resulted in a reduction of blood glucose and insulin resistance, improvement of β-cell function, total and LDL cholesterol levels. On the contrary, 25% of participants in the Placebo group have developed T2DM [111]. However, a randomised trial, designed in a similar condition in Australia (except for the zinc dose—30 mg of zinc gluconate daily) [112], did not support the beneficial effects of zinc supplemental in populations with a Western diet [113].

In total, zinc could be considered as an important marker of a healthier diet and/or lifestyle, associated with reduced risk of DM development. However, it is definitively not the key element, responsible for DM development and progression, and other factors, such as initial status of the target population (zinc replete or zinc deficient), prevalent diet and lifestyle, should be considered.

## 9. Conclusions

Mitochondria are the central player in the DCM research and development of the novel therapeutics for DCM treatment, as their dysfunction appears in the early stages of the DCM development. Maintenance of proper mitochondrial integrity, adequate balance between mitochondrial biogenesis, dynamics and mitophagy are crucial to maintain Ca^2+^ metabolism and a sufficient level of ATP production during DM-mediated metabolic alterations. Although in the past decade the certain advancement has been made in the understanding of the molecular mechanisms of DCM development, many questions remained unanswered and required more systematic biomedical and pharmaceutical research, preclinical and clinical investigations to develop the better treatments to prevent and treat the cardiovascular complications in diabetic patients.

## Data Availability

Not applicable.

## Abbreviations

ACC2	acetyl-CoA carboxylase 2
AF	atrial fibrillation
AKAP121	A-kinase anchor protein
ANP	atrial natriuretic peptide
Atg7	Autophagy-Related Protein 7
BNIP3	Bcl-2/adenovirus E1B 19-kDa protein-interacting protein 3
BNP	brain natriuretic peptide
BRD4	Bromodomain Containing 4
DCM	Diabetic cardiomyopathy
DM	diabetes mellitus
DNM1L	Dynamin 1 like
Drp1	Dynamic relative protein 1
eNOS	endothelial nitric oxide synthase
ETC	electron transport chain
FAs	fatty acids
FIS1	fission, mitochondrial 1
FoxO1	forkhead box protein O 1
GLUT4	glucose transporter type 4
Grp75	Stress-70 Protein, Mitochondrial
HF	high fat
HG	high glucose
IGFBP7	Insulin Like Growth Factor Binding Protein 7
IP3R	Inositol 1,4,5-Trisphosphate Receptor Type 3
LACS1	long chain acyl-CoA synthetase 1
LC3	Autophagy-Related Ubiquitin-Like Modifier LC3 A
LCAD	Acyl-CoA Dehydrogenase Long Chain
LTCCs	L-type calcium channels
LV	left ventricular
MAMs	mitochondrial associated membranes
MCU	mitochondrial Ca^2+^ uniporter
MFN1, MFN2	mitofusin 1 and 2
MICU1	mitochondrial calcium uptake 1
MMPs	matrix metalloproteinases
Mst1	Mammalian sterile 20-like kinase 1
OPA1	optic atrophy protein 1
Orai1	Ca^2+^ release-activated calcium channel protein 1
Parkin	Parkin RBR E3 Ubiquitin Protein Ligase
PDC	pyruvate dehydrogenase complex
PGC1α	Peroxisome proliferator-activated receptor gamma coactivator 1-alpha
PINK1	PTEN Induced Kinase 1
Rab9	Ras-related protein Rab-9
ROS	reactive oxygen species
SR	sarcoplasmic reticulum
STZ	streptozotocin
TGF-β1	Transforming growth factor beta 1
TSP-1	thrombospondin-1
Ulk1	unc51 like kinase 1
VDAC	Voltage-dependent anion-selective channel protein 1
ZDF	Zucker diabetic fatty
β-MHC	β-myosin heavy chain
